# The Grow It! app—longitudinal changes in adolescent well-being during the COVID-19 pandemic: a proof-of-concept study

**DOI:** 10.1007/s00787-022-01982-z

**Published:** 2022-05-07

**Authors:** E. Dietvorst, J. S. Legerstee, A. Vreeker, S. Koval, M. M. Mens, L. Keijsers, M. H. J. Hillegers

**Affiliations:** 1 Department of Child and Adolescents Psychiatry/Psychology Erasmus MC Sophia, Children’s Hospital, Erasmus University Medical Center, Rotterdam, The Netherlands; 2 Department of Epidemiology, Harvard T.H. Chan School of Public Health, Boston, USA; 3 Department of Psychology, Education and Child Studies, Erasmus School of Social and Behavioural Sciences, Rotterdam, The Netherlands

**Keywords:** Experience sampling method, Serious game, e-health, Internalizing problems, Prevention

## Abstract

Adolescent mental health and well-being have been adversely impacted by the COVID-19 pandemic. In this preregistered longitudinal study, we evaluated whether adolescents’ well-being improved after playing the multiplayer serious game app Grow It! During the first lockdown (May–June 2020), 1282 Dutch adolescents played the Grow It! app (age = 16.67, SD = 3.07, 68% girls). During the second lockdown (December–May 2020 onwards), an independent cohort of 1871 adolescents participated (age = 18.66, SD = 3.70, 81% girls). Adolescents answered online questionnaires regarding affective and cognitive well-being, depressive symptoms, anxiety, and impact of COVID-19 at baseline. Three to six weeks later, the baseline questionnaire was repeated and user experience questions were asked (*N* = 462 and *N* = 733 for the first and second cohort). In both cohorts, affective and cognitive well-being increased after playing the Grow It! app (*t* = − 6.806, *p* < 0.001; *t* = − 6.77, *p* < 0.001; *t* = − 6.12, *p* < 0.001; *t* = − 5.93, *p* < 0.001; Cohen’s *d* range 0.20–0.32). At the individual level, 41–53% of the adolescents increased in their affective or cognitive well-being. Adolescents with higher risk profiles (i.e., more depressive symptoms, lower atmosphere at home, and more COVID-19 impact) improved more strongly in their well-being. Positive user evaluations and app engagement were unrelated to changes in affective and cognitive well-being. This proof-of-concept study tentatively suggests that Grow It! supported adolescents during the pandemic.

**Supplementary Information:**

The online version contains supplementary material available at 10.1007/s00787-022-01982-z.

## Introduction

Mental health is broadly defined as the presence of positive indicators and the absence of negative indicators such as depressive symptoms or anxiety [1]. Well-being, in turn, entails both cognitive appraisal of life satisfaction (cognitive well-being) as well as affective components such as happiness (affective well-being) [2]. Investing in well-being may, as such, improve mental health.

Already before the COVID-19 pandemic, the need for psychological and psychiatric professional services outweighed the resources, resulting in long waiting lists [3]. COVID-19-induced social restrictions, such as homeschooling, social distancing, and limited opportunities for sports and leisure, have negatively impacted adolescent well-being and mental health [4–8]. Moreover, there is growing evidence on health-related outcomes such as decreases in physical activity during quarantine that may have contributed to an increase in adolescent mental health problems and depressive symptoms [9, 10], all in all making the shortage of professional services even more pressing [10]. Mobile Health (mHealth) can improve early identification, prevention, and treatment of mental health problems [11, 12], without being limited to professional resources: it is scalable and accessible, as adolescents can use mHealth interventions whenever and wherever they want. Moreover, feasibility studies suggest that adolescents perceive mHealth as less stigmatizing compared to face-to-face health care [13]. Even though several tools are currently available, the effects of online support are still poorly understood [14].

Grow It! has been developed for adolescents aged 12–25 years to improve adolescents’ affective and cognitive well-being, and consists of two components. Firstly, adolescents monitor their emotions and behaviors in daily life by utilizing the experience sampling method (ESM). This reporting on emotions at several random times per day may increase reflection and self-insights [15]. Second, Grow It! teaches how to cope with setbacks and promotes emotional resilience by offering daily challenges. As cognitive behavioral therapy (CBT) is one of the most effective interventions widely applied in psychological (preventive) care [16–18], the daily challenges are based on CBT and are aimed at strengthening adaptive coping, supporting physical activation and preventing emotional problems. The smartphone app, game mechanics and the daily challenges were co-created by adolescents, child psychologists/psychiatrists, researchers, and game designers. The developmental process, feasibility, and acceptability of the Grow It! app have been described elsewhere [19].

The Grow It! app was first launched during the COVID-19 pandemic. The current preregistered study tested four research questions: (1) first, we tested whether adolescents’ affective and cognitive well-being increased after playing the Grow It! app. We further described (2) how many adolescents increased in their affective and cognitive well-being. Finally, we explored (3) to which extent individual background characteristics and (4) user evaluations and engagement were associated with changes in affective and cognitive well-being.

## Methods

### Procedure and participants

**Fig. 1:**
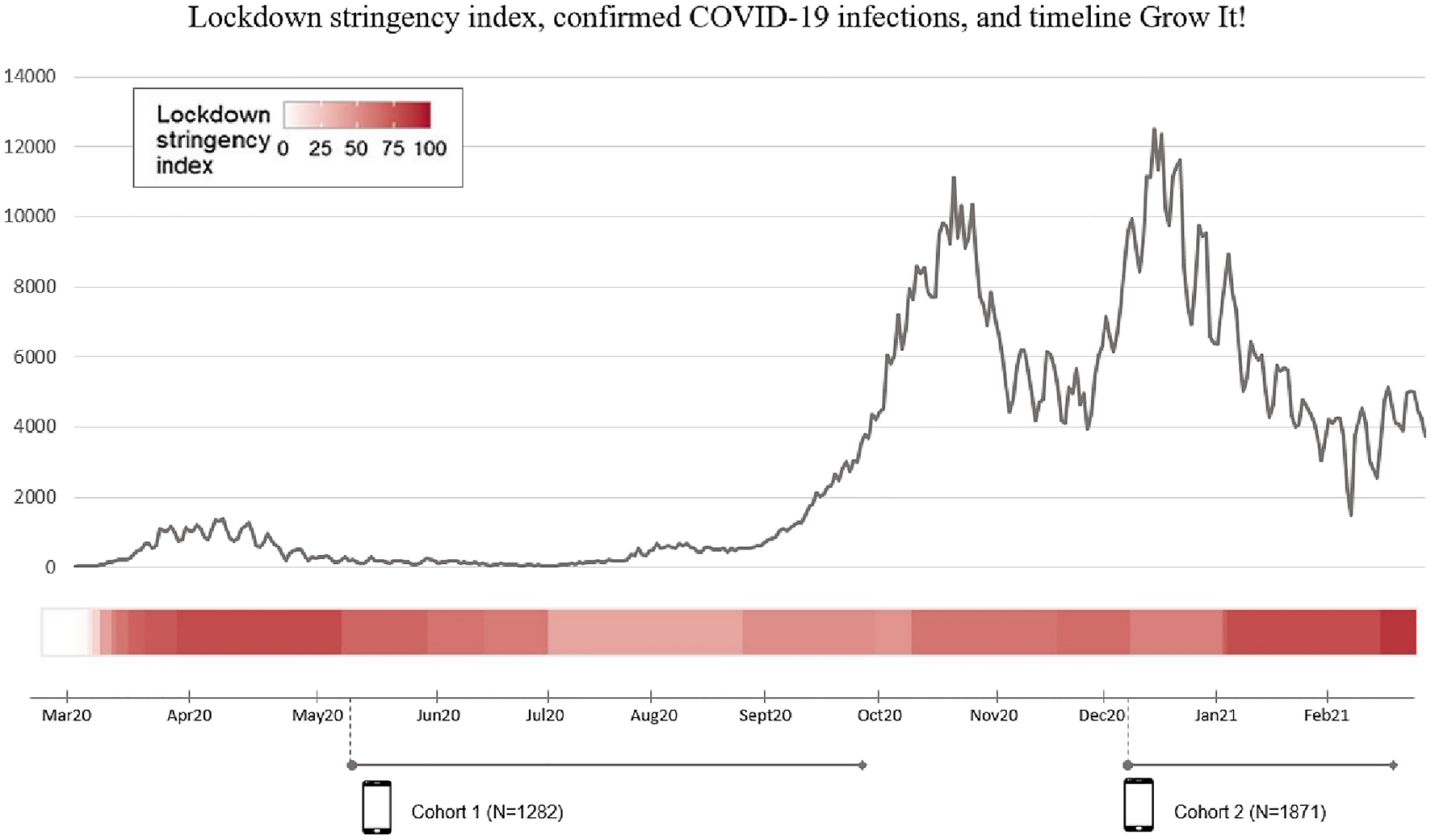
Timeline grow it!

In May 2020, during the first lockdown, 1282 adolescents (cohort 1: mean age 16.67 years, SD = 3.07, 68% girls) played the Grow It! app for 6 weeks (Fig. 1). In December 2020, during the second lockdown, another 1,871 adolescents (cohort 2: mean age 18.66 years, SD = 3.70, 81% girls) played the app for 3 weeks. To participate, adolescents needed to be able to read and write Dutch, live in the Netherlands, and own a smartphone. Non-probability convenience sampling took place via advertisements and a promotion video on (social) media and through online announcements by schoolteachers. All study information was published on our website, where it was also possible to contact the team of researchers, and sign the informed consent on a secure webpage for participants and also for parents if the age of the child was < 16 years. After the online baseline questionnaire (codebook), participants received an SMS with a unique login code to activate the app. Table 1 shows sample characteristics, Table 2 shows descriptive statistics of all study variables.

**Table 1:** Sample characteristics

	Cohort 1 (*N* = 1282)	Cohort 2 (*N* = 1871)
Demographics		
Age, years	16.67 (3.42)	18.66 (3.70)
Sex % girls	68%	81%
Education level^a^	5% p.s. 16% low 24% middle 55% high	1% p.s. 17% low 32% middle 39% high 10% other
Cultural identity	92% Dutch 7% mixed 1% other	97% Dutch 2% mixed 1% other
Mental health status		
In treatment for psychological problems (%)	14% (3% waiting list)	21% (5% waiting list)
Mode of contact since COVID-19 with psychologist/psychiatrist (%)	62% online 33% offline 5% none	33% online 65% offline 2% none

^**a**^p.s. = primary school, low = (preparatory school for) technical and vocational training, middle = (preparatory school for) professional education, high = (preparatory school for) university

**Table 2:** Study outcomes

	Cohort 1		Cohort 2	
	Baseline (*N* = 1282)	Follow-up (*N* = 462)	Baseline (*N* = 1871)	Follow-up (*N* = 733)
	Mean (SD)	Mean (SD)	Mean (SD)	Mean (SD)
Well-being				
Affective well-being	4.90 (1.35)	5.33 (1.29)	4.28 (1.40)	4.59 (1.38)
Cognitive well-being	6.46 (2.20)	7.12 (2.13)	5.50 (2.20)	5.95 (2.19)
Depressive symptoms	5.71 (4.33)	5.12 (4.08)	7.35 (4.44)	6.65 (4.16)
Anxiety symptoms	16.24 (4.83)	16.15 (4.73)	18.80 (4.48)	18.20 (4.51)
Coping strategies				
Adaptive coping	4.24 (1.37)	**–**	4.01 (1.27)	**–**
Maladaptive coping	3.38 (1.60)	**–**	3.56 (1.53)	**–**
Impact of COVID-19				
Difficulties with cancelations	2.48 (1.06)	**–**	2.58 (1.18)	**–**
Financial problems	1.30 (0.68)	**–**	1.32 (0.70)	**–**
Optimism about the future	2.22 (1.00)	**–**	1.84 (0.89)	**–**
Atmosphere at home	5.26 (1.24)	**–**	4.92 (1.41)	**–**
COVID19 stringency index	70.24 (2.79)	**–**	78.50 (2.46)	**–**
User evaluation and engagement				
User evaluation Grow It!	**–**	7.14 (1.45)	**–**	7.17 (1.32)
Challenges (%)	**–**	25.67 (26.78)	**–**	32.78 (33.39)
ESM compliance (%)	**–**	14.20 (20.27)	**–**	20.56 (25.79)

The Grow It! study was conducted in accordance with the guidelines proposed in the World Medical Association Declaration of Helsinki and has been approved by the Medical Ethical Committee of the Erasmus Medical Centre (registration number: MEC2020-0287).

### Description of the Grow It! app

**Fig. 2:**
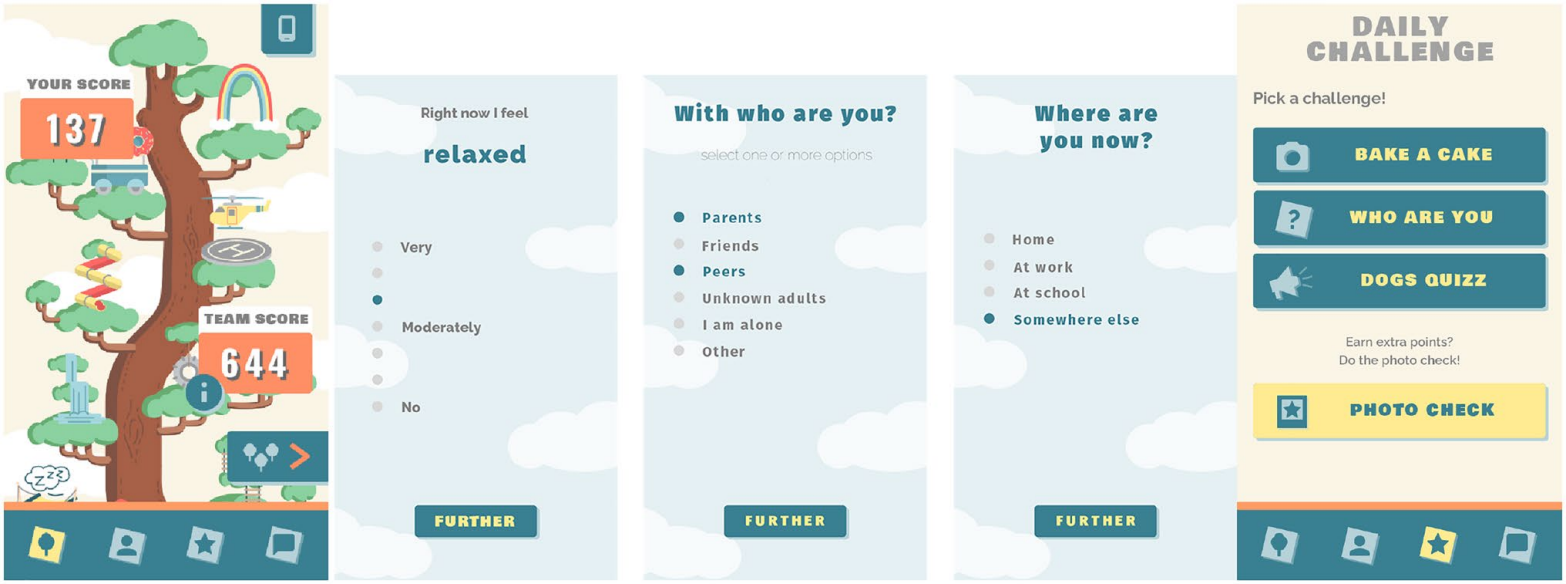
Screenshots Grow It! app

The Grow It! application is a multiplayer serious gaming smartphone tool (Android/iOS) aimed at promoting well-being (Fig. 2). Participants are randomly allocated to a team of four to six players. Within the app, each team collaborates in nurturing and embellishing their virtual tree, which grows each time the team reaches a predefined number of points (‘spurt’). Individual players earn points by filling out ESM questionnaires five random times per day between 9:00 and 21:00 and by completing daily challenges aimed at promoting adaptive coping including: social support, acceptance, problem-solving, and distraction. For instance, take a picture of a red car (photo challenge: aimed at distraction), ask someone what they like about you and write it down (assignment: aimed at social support), or ask someone whether he/she is having difficulties with and come up with a solution together. Write it down (assignment: aimed at problem solving)! Every day, adolescents can choose one out of three challenges. To demonstrate the successful accomplishment of a challenge, adolescents can either upload pictures, answer a free text form, or select the right answer option. Team members can communicate and motivate each other by means of positive stickers (chat function). Its privacy and security were approved by the privacy and security office of Erasmus MC, and the app complies with the Dutch General Data Protection Regulation (GDPR) and NEN-norm 7510:2017 (Dutch standard of information security management systems in healthcare).

### Measures

#### Well-being

As primary outcome, well-being was assessed at baseline and follow-up using two items [2]. The first item refers to affective well-being: “How happy did you feel in the past week?” rated on a seven-point scale ((1) not at all to (7) very much). Previous work shows good convergent validity of this single items measurement of affective well-being [19]. The second item assessed cognitive well-being: “How satisfied were you with your life in the past week?” and was rated on a ten-point scale ((1) not at all to (10) very much). The single item measure of cognitive well-being has been demonstrated to have good reliabilities that are comparable to multiple-item scales [20].

#### Depressive symptoms

Depressive symptoms were measured at baseline and follow-up with the short 12-item Children’s Depression Inventory questionnaire (CDI [21]). Each item has three statements from which the adolescent could choose the one that would best describe him/her over the last week. For example: ‘I am sad sometimes’ (0), ‘I am often sad’ (1), and ‘I am sad all the time’ (2). Total scores ranged from 0 to 24, with higher scores indicating more depressive symptoms. Cronbach’s α was good in cohort 1 and cohort 2 (Baseline: 0.84, 0.84 Follow-up: 0.84, 0.83) and in previous work (*α* = 0.80) [21].

#### Anxiety symptoms

Anxiety was measured at baseline and follow-up with the nine items of the Generalized Anxiety Disorder scale from the Screen for Child Anxiety Related Disorders (SCARED child version [22]). Participants were asked to choose one response that would best describe their feelings in the last 2 weeks. Example items are: “In the last 2 weeks I was nervous”, “In the last 2 weeks, I was worried whether other people would like me”. The response scale runs from “not at all” (0), “a little bit or sometimes” (1) to “definitely or often” (2). High scores on the SCARED indicate more anxiety. Cronbach’s α was good in cohort 1 and cohort 2 (Baseline: 0.88, 0.85 Follow-up: 0.89, 0.86) and comparable to previous research (*α* = 0.90) [23].

#### Impact of COVID-19

To measure the impact of COVID-19, we used three items from the CoronaVirus Health Impact Survey Questionnaire [24] tapping into difficulties with cancellations of (important) events, financial problems, and optimism about the future. Items are scored on a five-point Likert scale (1 = not at all, 3 = quite, 5 = very much). Moreover, to assess the atmosphere at home since COVID-19 participants were asked to rate the atmosphere at home from 1 = uncomfortable/not pleasant at all to 4 = a little bit pleasant and 7 = very comfortable/pleasant. As an indication of governmental restrictions related to COVID-19, we calculated the COVID-19 stringency index ([25]; 0–100).

#### User information of Grow It! app

Following the guidelines to measure user experience [26], evaluation of the app was measured at follow-up by asking participants what grade they would give to the Grow It! app, with an answer scale from 1 to 10. Also, participants were asked whether they would recommend the Grow It! app to their friends and what kind of effect the app had on their behavior and feelings. App engagement was operationalized in terms of the percentage of completed daily challenges in the Grow It! app (0–100%) and the compliance of the ESM is the percentage ESM questionnaires that were completed in the Grow It! app (0–100%) [27].

##### Education level

One item in the baseline questionnaire assessed adolescent education level (primary school, low = (preparatory school for) technical and vocational training, middle = (preparatory school for) professional education, high = (preparatory school for) university).

### Statistical analyses

Before testing our hypotheses, we ran tests for cohort -differences (Appendix A1) and attrition (Appendix A2). Because participants between cohort 1 and cohort 2 differed on sex, mean age, cultural identity and COVID-19 stringency index, hypotheses were tested separately for cohort 1 and cohort 2 and sensitivity analyses were added to assess the effects of attrition (Appendix A3). To assess our first hypothesis (1), we conducted paired samples t tests to compare the affective and cognitive well-being from baseline to follow-up. To determine (2) how many adolescents increased in well-being, we followed a method by Grice and colleagues on persons and effect sizes [26]. We considered an individual change of 0.2 SD (small effects size according to Cohen’s D) as a meaningful increase or decrease [28–32]. To understand (3) how background characteristics and (4) user experience and app engagement (challenges and compliance) would be associated with changes in affective and cognitive well-being after playing the Grow It! app, we had preregistered ANOVA and correlations with change rates. Here, we improved our analytical plan and conducted repeated measure models for each predictor (between * within effect). To correct for multiple testing, for each research question separately, the false discovery rate (FDR) was applied [33]. All statistical calculations were performed using IBM SPSS Statistics (version 25).

## Results

**Fig. 3:**
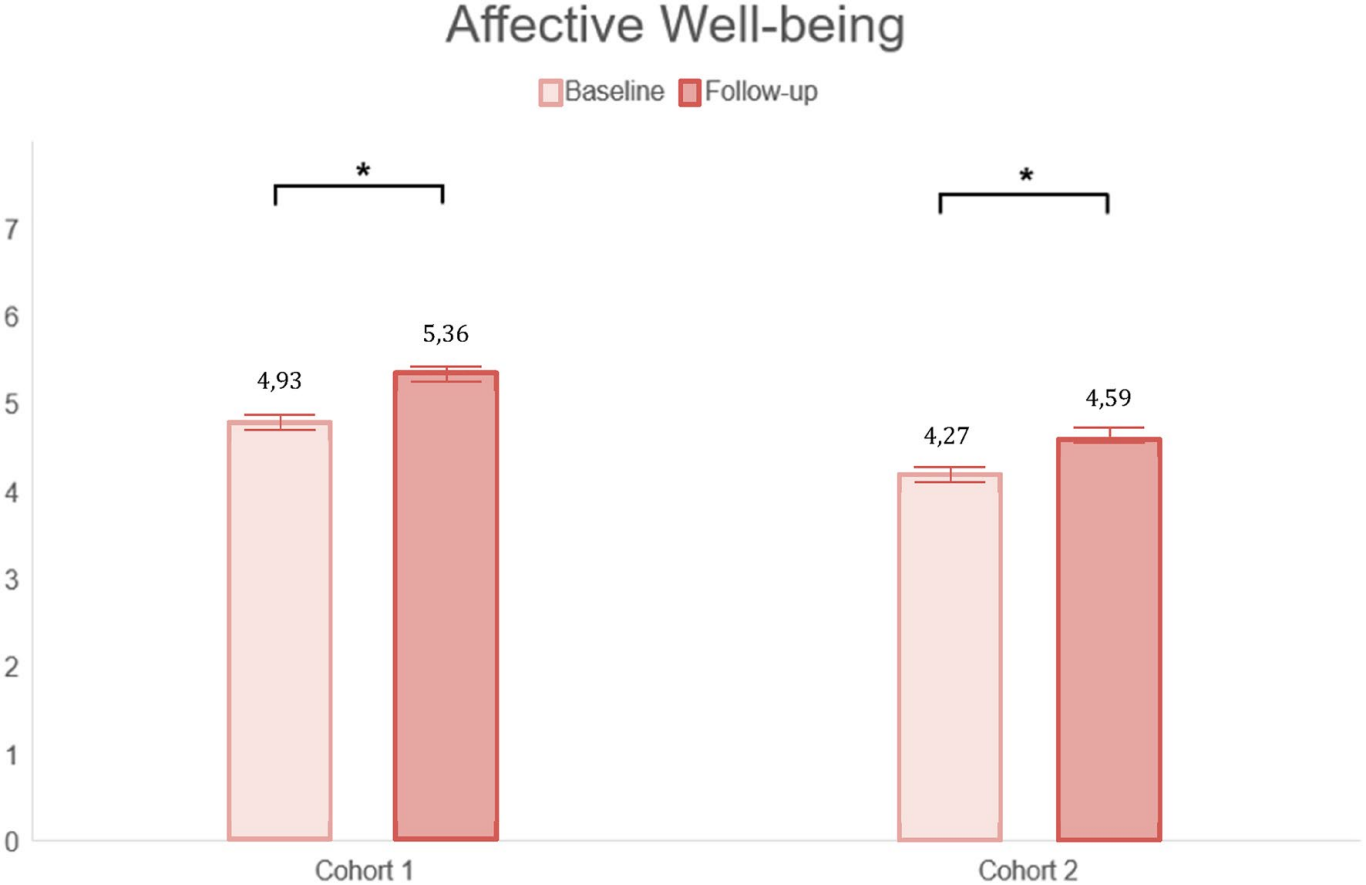
Changes in affective well-being

**Fig. 4:**
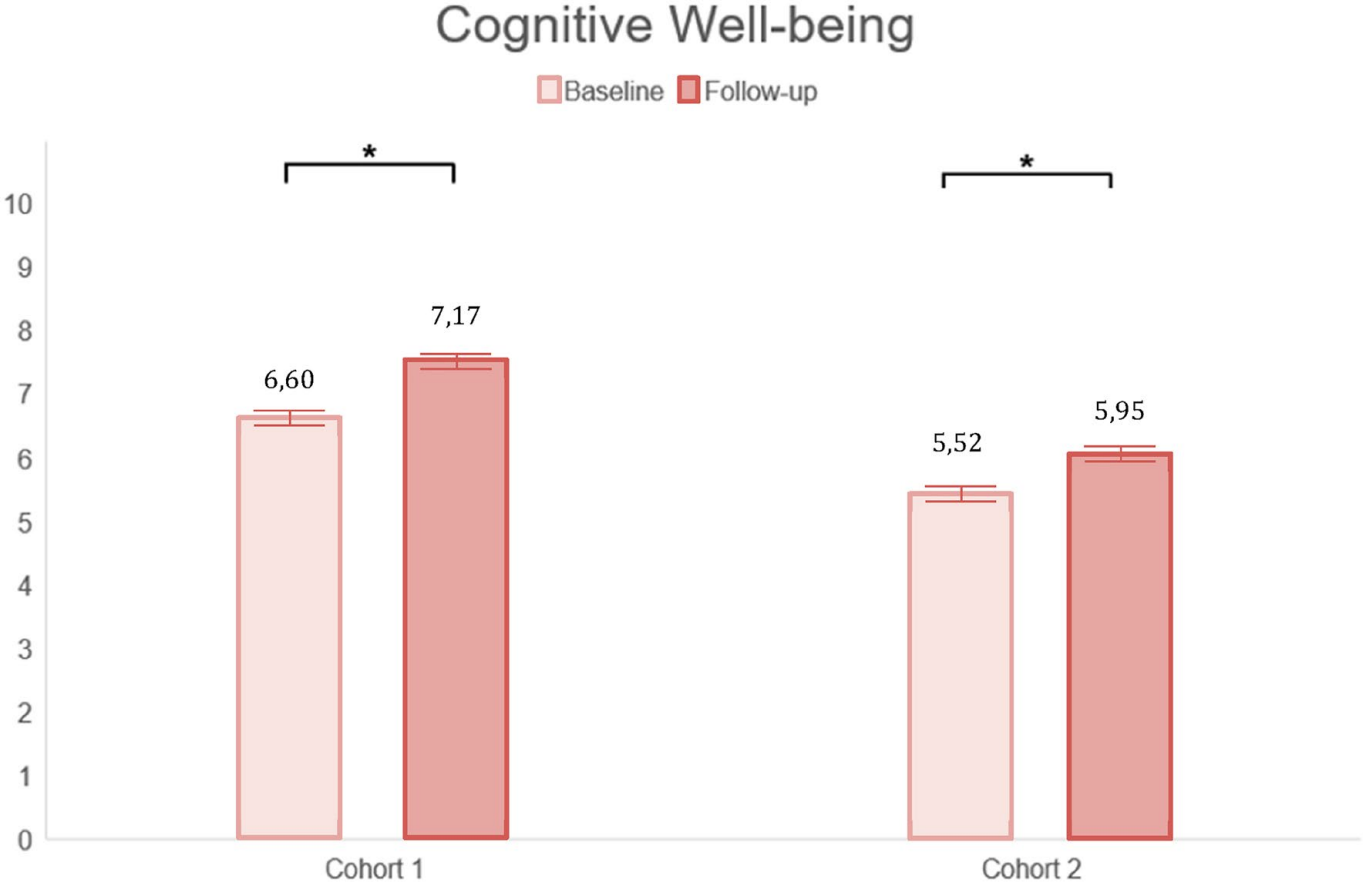
Changes in cognitive well-being

After playing the Grow It! app, affective well-being statistically increased from baseline to follow-up in both cohort 1 (*t*(461) = − 6.806, *p* < 0.001, *d* = 0.32) and in cohort 2 (*t*(732) =  − 6.77, *p* < 0.001, *d* = 0.23). Similarly, cognitive well-being statistically improved from baseline to follow-up in cohort 1 (*t*(461) = − 6.12, *p* < 0.001, *d* = 0.27) and cohort 2 (*t* (732) =  − 5.93, *p* < 0.001 *, *d* = 0.20; see Table 3 and Figs. 3, 4). Exploratory sensitivity analyses were carried out in a regression framework to rule out confounders (gender, age, education level, COVID-19 stringency), which yielded similar results (Appendix A3). Together, these results confirm our first hypothesis. In addition, we explored changes in two other indicators of well-being, namely symptoms of depression and anxiety. Depressive symptoms significantly decreased from baseline to follow-up in both cohorts (cohort 1: *t*(461) = − 2.91, *p* = 0.004*, *d* = 0.08, cohort 2: *t* (732) = − 7.34, *p* < 0.001, *d* = 0.17). Moreover, anxiety significantly decreased in cohort 2 (*t* (732) = − 4.69, *p* < 0.001, *d* = 0.14), but not in cohort 1. Notably, the effect sizes for the significant decreases in depressive symptoms and anxiety symptoms are small. Looking at the individual level, 41–53% of the adolescents increased meaningfully in their cognitive and affective well-being (*d*: ≥ 0.2), respectively, from baseline to follow-up. More specifically, in cohort 1, 45% adolescents increased, 21% decreased (*d*: ≤ 0.2), and 34% remained stable in their affective well-being. In cohort 2, 42% increased, 23% decreased, 35% remained stable in affective well-being. Furthermore, 53% increased, 26% decreased and 21% remained stable in cognitive well-being in cohort 1. In cohort 2, 45% increased, 30% decreased, and 25% remained stable in cognitive well-being. Each group is described in detail in Appendix A4-A7.

**Table 3:** Changes in well-being before and after Grow It!

	Cohort 1 (*N* = 462)				Cohort 2 (*N* = 733)			
	Overall change^a^	*t*-test	*p*	Effect size (*d*)	Overall change	*t*-test	*p*	Effect size (*d*)
Primary outcomes^b^								
Affective well-being	+ 0.42	− 6.81	< 0.001*	0.32	+ 0.32	− 6.77	< 0.001*	0.23
Cognitive well-being	+ 0.57	− 6.12	< 0.001*	0.27	+ 0.43	− 5.93	< 0.001*	0.20
Secondary outcomes^c^								
Depressive symptoms	− 0.35	− 2.91	0.004*	0.08	− 0.75	− 7.34	< 0.001*	0.17
Anxiety symptoms	− 0.06	0.43	0.671	0.01	− 0.63	− 4.69	< 0.001*	0.14

*Corrected alpha was 0.004

^a^Overall change (mean follow-up – mean baseline)

^b^Preregistered outcomes

^c^Exploratory outcomes; not preregistered

**Fig. 5:**
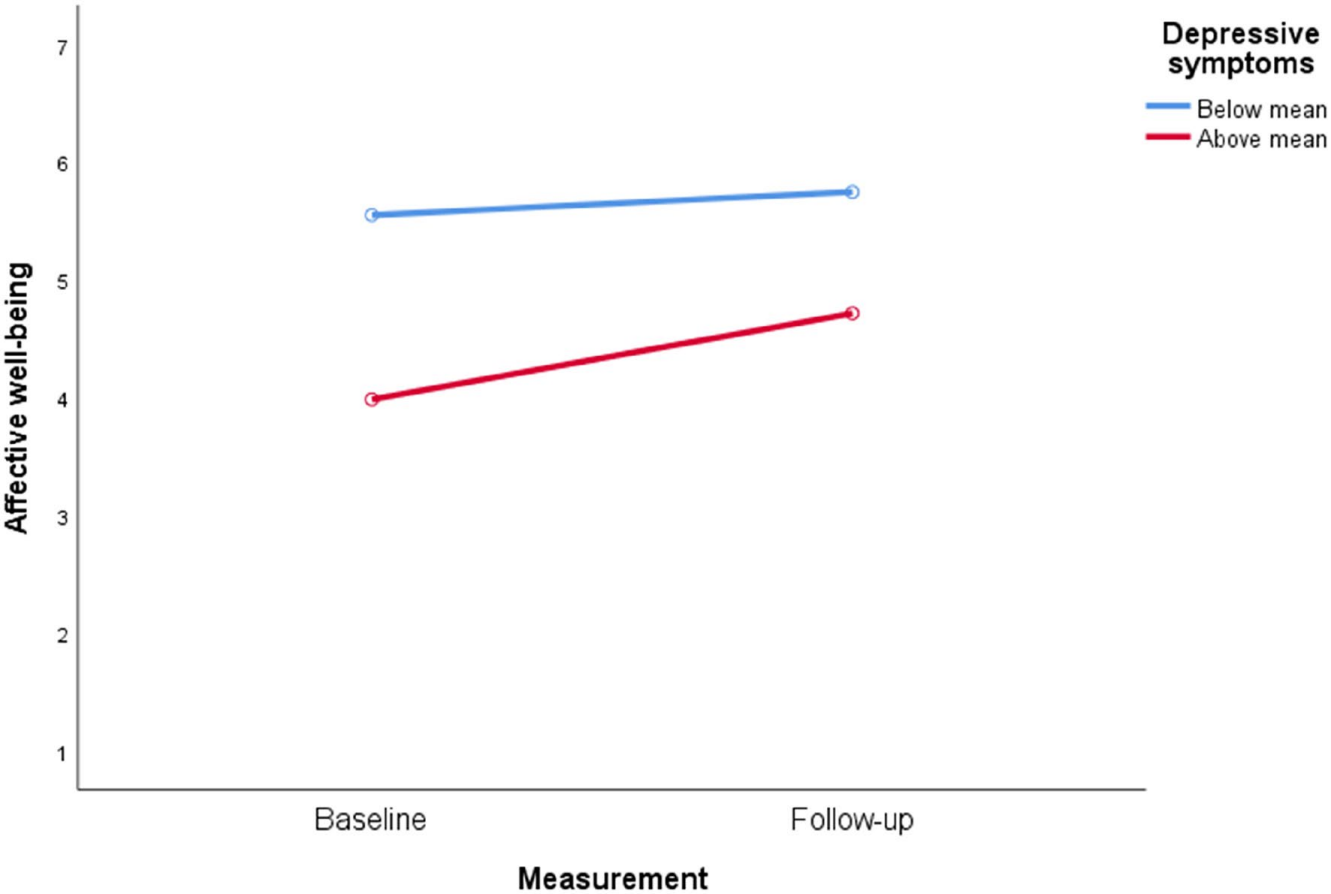
Illustration of difference in well-being from baseline to follow-up separated for adolescents with depressive symptoms below and above mean (cohort 1)

Table 4 shows the results of repeated measures ANOVA to test whether within-person changes in affective or cognitive well-being over time, would be related to between-person differences in demographics, mental health status, coping strategies and impact of COVID-19. Except for an association with education level in cohort 1, none of the demographics (sex, age, cultural identity) were related to changes in well-being. Mental health status was unrelated to changes of well-being. However, stronger increases in well-being were found for adolescents with higher baseline scores of depression (cohort 1–2, see Fig. 5) and among more anxious adolescents (cohort 1). More positive changes in well-being were further associated with a stronger impact of COVID-19 (i.e., more financial problems, lower optimism about the future, lower atmosphere at home (cohort 1), and more difficulties with cancelled events (cohort 2). Finally, changes in well-being were more positive with a lower COVID-19 stringency (i.e., fewer lockdown measures; cohort 1). In contrast to our preregistered hypotheses, neither more positive evaluations nor more engagement (compliance and challenges) were related to the strength of changes over time in affective and cognitive well-being.

**Table 4:** Moderators of changes in affective and cognitive well-being

	Cohort 1 (*N* = 462)		Cohort 2 *(N* = 733)	
	Change^a^ affective well-being	Change cognitive well-being	Change affective well-being	Change cognitive well-being
Demographics				
Sex	0.64	0.27	5.18	2.65
Education	0.64	7.27*	0.10	0.51
Age	0.81	1.47	0.01	0.08
Ethnicity	3.59	3.15	0.86	0.12
Mental health				
In treatment of psychological problems	4.97	0.50	0.86	1.72
Well-being				
Depressive symptoms	22.46*	9.14*	8.09*	7.25*
Anxiety symptoms	20.48*	12.49*	5.35	1.16
Coping strategies				
Adaptive coping	2.43	5.84	0.43	0.96
Maladaptive coping	4.73	0.11	0.00	0.04
Impact of COVID-19				
Difficulties with cancelations	1.53	1.00	6.59*	10.58*
Financial problem	8.82*	0.05	1.70	2.25
Optimism about future	8.12*	15.32*	1.25	1.58
Atmosphere at home	29.03*	10.19*	3.35	4.11
COVID-19 stringency index	16.72*	9.06 *	1.50	1.48
User evaluation and engagement				
User evaluation Grow It!	0.64	0.55	1.47	0.89
Challenges	0.97	1.41	0.42	0.48
ESM compliance	0.10	0.07	0.73	1.26

*Corrected alpha was 0.01

^a^Results are derived from repeated measures ANOVA with baseline and follow-up measure of well-being as repeated measures and time × moderator als cross-level interaction term. Test statistics displayed are *F*-statistics, with *p* values

## Discussion

This preregistered study presents the first evaluation on changes in well-being after playing the Grow It! app [18], a multiplayer mHealth tool which combines ESM with gamified CBT in a large community sample of adolescents carried out during the COVID-19 pandemic. Both cognitive and affective well-being increased significantly during the course of the study—and symptoms of depression and anxiety significantly decreased. Changing COVID-19 related restrictions could not explain this result. Between 41 and 53% of the adolescents improved meaningfully in their cognitive and affective well-being (> 0.2 SD over 3 or 6 weeks of Grow It! app engagement). Overall, improvements in cognitive and affective well-being were stronger among adolescents with higher risk profiles in terms of baseline emotional problems, home context, and COVID-19 impact, and under circumstances with fewer lockdown measures. Grow It! Players’ engagement and satisfaction were unrelated to improvements in well-being. Even though it is not yet possible to draw firm conclusions regarding the effectiveness of the app with this study, our findings provide the first tentative insights that well-being improves and depressive and anxiety symptoms decrease: a proof of concept.

### Changes in affective and cognitive well-being after playing the Grow It! app

Grow It! is based on two elements, which have proven successful in reducing mental health problems in face-to-face clinical practice, self-monitoring and insight of emotions and CBT [15–19]. Already after using the Grow It! app for 3 or 6 weeks, the majority of participating adolescents improved in their affective and cognitive well-being (41–53%)—in a period in which the overall well-being of adolescents in the general population decreased [6]. Without a control group, however, it is hard to establish how Grow It! has contributed to this change, and how the effects of COVID-19 and Grow It! upon well-being are intertwined. The study also noticed differences between participants. A group of adolescents did not change meaningfully (21–35%) and a smaller group decreased in their well-being (< 0.2 SD, 21–30%).

Adolescents who reported more depressive symptoms (cohort 1–2) or anxiety symptoms (cohort 1) at baseline increased more strongly in their well-being over the course of our study. This suggests that self-monitoring emotions and being challenged toward adaptive coping, as Grow It! does, is particularly helpful for those at risk. Although mHealth will probably never replace a professional, it could become a helpful add-on tool to already promote self-reflection and activate coping in times of being on a waiting list, or in a blended approach during treatment [31]. Future research will have to determine how Grow It! can help to prevent mental health problems among other high-risk populations, such as chronically ill children or children of parents with mental illness (COPMI) [13].

Even though this study describes a large longitudinal study with pre-registered analyses, the presented findings here should be interpreted in light of several limitations. As the COVID-19 pandemic has drastically disrupted the daily lives of all young people in the Netherlands [3–8], we decided not to add a control group to our study but instead make the app available to every young Dutch person aged 12–25 years. Moreover, as the current study started during COVID-19, we had no pre-COVID data from participants. Future research is needed, preferably with a Randomized controlled trial (RCT) design, in which the influence of societal circumstances can be separated from effects of the Grow It! app. This way, alternative explanations of our results cannot definitely be ruled out. Currently, an RCT to better understand the effectiveness of the Grow It! app among adolescents at risk for emotional problems is ongoing. Retention rates (35.41% of *n* = 1282 in cohort 1, 39% of *n* = 1871 in cohort 2) were suboptimal, but comparable with other full online youth intervention studies [34] while they were influenced by the fact that participants were not paid and that the study on the Grow It! was carried out during a crisis with no possibility of face-to-face contact to motivate participants. Our nationwide approach to promote Grow It! through (social) media also led to an oversampling of girls, adolescents who have a smartphone, adolescents who are often engaged in online behavior, adolescents with higher education level, as well as adolescents in treatment for mental health problems. This suggests the presence of sampling bias and therefore limits the generalizability of our results to the population. It also informs us that this may be the population that is most interested, motivated for and in need of mHealth support [35]. Whether indeed the Grow It! app may work as a targeted preventive intervention tool especially for adolescents at risk for mental health problems and what the individual underlying working mechanisms are remain important topics of further research.

### Lessons learned and consequences for the future

Carrying out a study among adolescents during the COVID-19 pandemic has taught us several important lessons. 1. We were able to reach many adolescents who had an interest in their emotional wellbeing and in strengthening their resilience in times of stress. 2. Since adolescents spend several hours per day online [36], online study recruitment (e.g., through social media, influencers), inclusion (e.g., through video-calls), and datacollection (e.g., with smartphone apps) turned out to be a feasible approach which integrates very well into their daily live. 3. Moreover, not only adolescents were eager to use the Grow It! app – parents, teachers and healthcareprofessionals had a great interest in offering our supportive Mhealth application to the young in this stressful situation. In the past two years COVID-19 has impacted the well-being of almost all adolescents, but youth at high risk for psychological problems (i.e., adolescents with parental psychopathology or with a chronic illness) might specifically benefit from this Mhealth support tool. Future preventive intervention studies will focus on these high risk populations. As a society, one way to take care of each other is to invest in scalable interventions to prevent adolescent mental health problems. Currently there are several Mhealth tools available and advice on how to improve mental health is omnipresent through social media, but very few interventions or advice are grounded in solid scientific research and are developed by independent parties who put the interests of adolescents first. In sum, mental health of adolescents is a crucial societal resource, which can be guarded through investing in scientifically based, user-friendly online support for adolescents.

## Conclusion

After 3 or 6 weeks of playing Grow It!, a multiplayer game based on self-monitoring (ESM) and CBT-based challenges, adolescents’ cognitive and affective well-being had significantly increased. Overall, 41–53% of the adolescents had meaningful increase in their affective or cognitive well-being during our study. Positive effects appeared stronger for adolescents with a high-risk profile at baseline (i.e., more depressive symptoms, lower atmosphere at home, higher COVID-19 impact). Even though the findings will need to be replicated in an RCT, these first findings suggest that Grow It! has contributed to adolescent well-being during COVID-19, especially among adolescents with higher risk profiles.

## Supplementary Information

Below is the link to the electronic supplementary material.

Supplementary file1 (DOCX 55 KB)

## Data Availability

Online codebook available at OSF https://osf.io/2at58/?view_only=b691104ecc3d45ad8b48e1bd60ad7125.

## Code availability

Syntax will be made public on https://osf.io/2at58/?view_only=b691104ecc3d45ad8b48e1bd60ad7125.
