# Supplementary material for: The Grow It! app—longitudinal changes in adolescent well-being during the COVID-19 pandemic: a proof-of-concept study
Source: Eur Child Adolesc Psychiatry. 2022 May 7;32(6):1097–107. doi: 10.1007/s00787-022-01982-z (PMC9076805; doi:10.1007/s00787-022-01982-z)
Supplement: Supplementary file 1 — Supplementary file1 (DOCX 55 KB) [file 787_2022_1982_MOESM1_ESM.docx]

# Supplementary Material (SI)

# Appendix A1. Missing data analyses

**Comparison between Cohort 1 and 2**

We have examined whether participants between Cohort 1 and 2 were comparable on their demographic characteristics and health care status. As this was not the case for proportion of girls (more girls in Cohort 2; χ2 (1) = 67.61 *p* =.000*), mean age (higher mean age in Cohort 2; *t* (3151) = 15.29, *p* = .000***) and cultural identity (higher proportion Dutch in Cohort 2; χ2 (2) = 37.78 p=.000*) and more stricter governmental measures at the time of the data collection of Cohort 2 (*t* (3151) = 87.80, *p =* .000*_, we have followed our preregistration and present the results for the main analyses separately for Cohort 1 and Cohort 2.

**Attrition bias and Missing data within Cohort 1 and Cohort 2**

In order to inspect whether there was attrition from baseline to follow up we have compared non-responders (n=828; n=1138) with responders (n=454; n=732) from Cohort 1 and 2 respectively with regard to primary outcome measures, demographic characteristics, and health care status (i.e., affective well-being, cognitive well-being, sex, age, cultural identity, education level, persons in the household, rooms in the household, (mental) health care status).

In Cohort 1, we have found no statistical differences between baseline and follow up on our primary outcome measures affective well-being (*t* (1280) = 0.64, *p* = .522) and cognitive well-being (*t* (1280) = 1.63, *p* = .103). Secondary well-being outcomes did also not differ statistically: depressive symptoms (*t* (1280) = 0.38, *p* = .706), anxiety symptoms (*t* (1280) = 50, *p* = .620), and loneliness (*t* (1280) = 1.23, *p* =.218). Furthermore no differences were found between non-responders and responders on cultural identity (χ2 (2) = 2.06 *p* =.357), person in the household (*t* (1280) = 1.17, *p =* .241), rooms in the household (*t* (1280) = 2.22, *p =* .027), having a chronically physical disease (χ2 (1) = 0.05, *p* =.818, in treatment of psychological problems (χ2 (2) = 5.37, *p* =.068) and mode of psychological treatment (χ2 (2) =1.10, *p* =.578). We did however detect significant differences when it comes to sex (χ2 (1) = 60.24 *p* =.000*), age (*t* (1280) = 3.17, *p =* 0.002*), education level ( χ2 (2) = 14.30 *p* =.003*) and COVID-19 Stringency Index (*t* (1280) = 2.97, *p =* 0.003*). That is, at follow-up the proportion of girls is higher, the mean age is lower, the proportion of highly educated adolescents is higher, and the strictness of governmental measures was less.

Likewise, in Cohort 2 we have found no statistical differences between baseline and follow up on our primary outcome measures affective well-being (*t* (1896) = 0.15, *p* = .880) and cognitive well-being (*t* (1896) = 0.29, *p* = .774). Secondary well-being outcomes did also not differ statistically: depressive symptoms (*t* (1869) = 0.19, *p* = .849) anxiety symptoms (*t* (1869) = 0.05, *p* = .963), and loneliness (*t* (1869) = 85, *p* = .396). Moreover, no differences were detected regarding sex (χ2 (1) = 1.22 *p* =.270), age (*t* (1869) = 1.65, *p =* .098), cultural identity (χ2 (2) = 7.33, *p* =.026), persons in the household (*t* (1869) = 0.27, *p =* .786), rooms in the household (*t* (1869) = 0.20, *p =* .845), being in treatment for psychological problems (χ2 (2) = 3.47, *p* =.176) and mode of psychological treatment (χ2 (2) = 3.28, *p* =.19383) and COVID-19 Stringency Index (*t* (1869) = 0.77, *p =* .439). We did however find differences in education level among the non-completers and the completers (χ2 (3) = 30.43, *p* =.000*). The proportion of highly educated adolescents is increased at follow-up.

In preparation for the main analysis, we have inspected whether data of baseline characteristics (i.e., age, sex, education, cultural identity) and study varaibles (affective and cognitive well-being, depressive symptoms, anxiety, and coping) was missing completely at random in Cohort 1 and Cohort 2. Also patterns of missing data of study variables at follow-up were inspected. In Cohort 1 86.2% of the baseline data and 77.5% of the follow-up data were present. For Cohort 2 88.0% of the baseline data and 87.0% of the follow-up data were available. In each of the Cohorts, the pattern of missing data was tested against the MCAR pattern using the Little MCAR test. Although the MCAR test showed a significant p-value in Cohort 2 at baseline, the Bollen/criterion (χ2/df < 3) suggests that the MCAR criterion was not seriously violated. (Bollen 1989) Cohort 1: 32.23/28=1.15 and 11.23/5=2.25; Cohort 2: 57.02/25=2.28; 3.96/5=0.79.

Relatedly, retention rates (35,41% of n=1282 in cohort 1, 39.12% of n=1871 in cohort 2) were comparable with other full online youth intervention studies [29] and were influenced by the fact that participants were not paid and that Grow It! was carried out for preventive use and therefore adolescents were not motivated by a burden of disease.

## Appendix A2. Table Differences within and between Cohort 1 and 2.

|  | **Cohort 1** | | | | |  | **Cohort 2** | | | | |  | **Differences baseline**  **Cohort 1 vs. Cohort 2 ^b^** |  |
| --- | --- | --- | --- | --- | --- | --- | --- | --- | --- | --- | --- | --- | --- | --- |
|  | **Baseline (*N*=1282)** |  | **Completers**  **(*N* =462)** | **Non-completers**  **(*N* =826)** | **Attrition** | ***p*** | **Baseline**  **(*N* =1871)** |  | **Completers**  **(*N* =732)** | **Non-completers**  **(*N* =1139)** | **Attrition** | ***p*** | **Baseline (*N* =1282,1871)** | ***p*** |
| **Demographics** | | | | | | | | | | | | | |  |
| Mean age | 16.67 (3.42) |  | 16.26  (3.07) | 16.89  (3.58) | *t* (1280) = 3.17 | .002* | 18.66  (3.70) |  | 18.77  (3.84) | 18.48  (3.46) | *t* (1869) = 1.65 | .098 | *t* (3151) = 15.29 | <.001* |
| Sex %girls | 67.6% |  | 73.7% | 64.1% | χ2 (1) = 60.24 | <.001* | 80.5% |  | 79.2% | 81.3% | χ2 (1) =  1.22 | .270 | χ2 (1) =  67.61 | <.001* |
| Education level^a^ | 4.5% p.s,  16.4% low, 24.5% medium  54.6% high* |  | 2.3% p.s.,  14.1% low,  24.2% medium, 59.4% high* | 5.8% p.s.,  17.9% low,  24.7% medium,  51.6% high* | χ2 (2) = 14.30 | .003* | 1.6% p.s.,  17.1% low,  32.1% medium, 39.1%high  10.1% other* |  | 2.0% p.s.,  18.4% low,  32.7 medium,  34.8% high  12.1% other* | 1.0% p.s.,  15.2% low,  31.2% medium,  45.5% high  7.1% other* | χ2 (3) =  30.43 | <.001* | n.a. |  |
| Cultural identity | 92.1% Dutch,  6.9% mixed  0.9% other |  | 90.7% Dutch, 8.1% mixed 1.1% other | 93.0% Dutch  6.2% mixed  0.8% other | χ2 (2) = 2.06 | .357 | 96.8%Dutch, 2.4%mixed 0.8%other |  | 96.0% Dutch  2.9% mixed  1.1% other | 98.1%Dutch, 1.7%mixed 0.3%other | χ2 (2) =  7.33 | .026 | χ2 (2) = 37.78 | <.001* |
| Persons in household | 3.36  (1.16)  0-16 |  | 3.41  (1.11)  0-16 | 3.33  (1.20)  0-16 | *t* (1280) = 1.17 | .241 | 3.38  (3.25)  0-200 |  | 3.41  (2.05)  0-36 | 3.37  (3.62)  0-200 | *t* (1869) = 0.27 | .786 | *t* (3151) = 0.21 | .830 |
| Rooms in household | 6.63  (3.02)  0-22 |  | 6.88  (3.21)  0-22 | 6.49  (2.90)  0-20 | *t* (1280) = 2.22 | .027 | 6.34  (7.57)  0-220 |  | 6.39  (3.25)  0-30 | 6.32  (9.35)  0-220 | *t* (1869) = 0.20 | .845 | *t* (3151) = 1.30 | .190 |
| **Mental health status** | | | | | | | | | | | | | |  |
| In treatment for psychological problems | 13.8%  (and 2.8% waitinglist) |  | 13.2%  (and 1.5% waitinglist) | 14.2%  (and 3.7% waitinglist) | χ2 (2) = 5.37 | .068 | 20.9%  (and 4.6% waitinglist) |  | 20.8%  (and 5.6% waitinglist) | 21.%  (and 3.8% waitinglist) | χ2 (2) =  3.47 | .176 | n.a. |  |
| Mode of contact since COVID-19 with psychologist/psychiatrist | 61.8% online  33.1l% offline  5.1% none |  | 65.0% online, 31.7%offline  3.3% none | 59.8% online,  34% offline,  6.2% none | χ2 (2) =  1.10 | .578 | 32.5% online 65.3% offline  2.2% none |  | 32.9% online 66.4% offline  0.7% none | 32.2% online  64.5% offline  3.3% none | χ2 (2) =  3.28 | .194 | n.a. |  |
| In treatment of chronically somatic disease | 10.8% |  | 10.6% | 11.0% | χ2 (1) = 0.05 | .818 | - |  | - | - | - |  | n.a. |  |
| Affective well-being | 4.90 (1.35) 1-7 |  | 4.93  (1.33)  1-7 | 4.88  (1.34)  1-7 | *t* (1280) = 0.64 | .522 | 4.28  (1.40)  1-7 |  | 4.27  (1.37)  1-7 | 4.28  (1.42)  1-7 | *t* (1896) = 0.15 | .880 | n.a. |  |
| Cognitive well-being | 6.46 (2.20) 1-10 |  | 6.60  (2.09) | 6.39  (2.26)  1-10 | *t* (1280) = 1.63 | .103 | 5.50  (2.20)  1-10 |  | 5.52  (2.10)  1-10 | 5.49  (2.27)  1-10 | *t* (1896) = 0.29 | .774 | n.a. |  |
| Depressive symptoms | 5.71 (4.33) |  | 5.62  (4.12)  0-20 | 5.71  (4.07)  0-20 | *t* (1280) = 0.38 | .706 | 7.35  (4.44)  0-24 |  | 7.37  (4.41)  0-23 | 7.33  (4.46)  0-24 | *t* (1869) = 0.19 | .849 | n.a. |  |
| Anxiety symptoms | 16.24 (4.83)  9-27 |  | 16.15  (4.66)  9-27 | 16.29  (4.93)  9-27 | *t* (1280) = 0.50 | .620 | 18.80  (4.48)  9-27 |  | 18.80  (4.42)  9-27 | 18.81  (4.53)  9-27 | *t* (1869) = 0.05 | .963 | n.a. |  |
| **Impact of COVID-19** | | | | | | | | | | | | | | |
| COVID-19 stringency index | 70.24 (2.78) |  | 69.93 (3.09) | 70.41 (2.59) | *t* (1280) = 2.97 | .003* | 78.50 (2.46) |  | 78.54 (2.37) | 78.45 (2.58) | *t* (1869) = 0.77 | .439 | *t* (3151) = 87.80 | <.001* |

Note: * indicates significant after multiple test correction following the FDR-derived significance threshold. 1. ^a^ p.s. = primary school, low =(preparatory school for) technical and vocational training, middle =(preparatory school for) professional education, high =(preparatory school for) university. ^b^ For group comparisons Independent Sample T-tests or χ2 tests are used.

## Appendix A3. Stepwise regression of Affective and Cognitive Wellbeing at follow-up

| **Model** | **Predictor** | **Beta** | ***p*** | **Adjusted R^2^** | ***p*** |
| --- | --- | --- | --- | --- | --- |
| **Cohort 1 [DV Affective well-being Follow-up]** | | | | | |
| 1 |  |  |  | 0.047 | <.001* |
|  | Sex | .14 | .003 | - | - |
|  | Age | -.16 | .001 | - | - |
|  | Education | .04 | .381 | - | - |
|  | COVID-19 index | -.05 | .262 | - | - |
| 2 |  |  |  | 0.262 | <.001* |
|  | Sex | .07 | .106 |  |  |
|  | Age | -.06 | .146 | - | - |
|  | Education | .04 | .299 | - | - |
|  | COVID-19 index | -.11 | .007 | - | - |
|  | Affective well-being Baseline | .49 | .000 | - | - |
| **Cohort 2 [DV Affective well-being Follow-up]** | | | | | |
| 1 |  |  |  | .009 | .007 |
|  | Education | -.10 | .007 | - | - |
| 2 |  |  |  | .314 | <.001* |
|  | Education | -.05 | .107 | - | -- |
|  | Affective well-being Baseline | .56 | .000 | - | - |
| **Cohort 1 [DV Cognitive well-being Follow-up]** | | | | | |
| 1 |  |  |  | 0.089 | <.001* |
|  | Sex | .18 | .000 | - | - |
|  | Age | -.19 | .000 | - | - |
|  | Education | .12 | .016 | - | - |
|  | COVID-19 index | .001 | .895 | - | - |
| 2 |  |  |  | 0.343 | <.001* |
|  | Sex | .11 | .005 | - | - |
|  | Age | -.06 | .151 | - | - |
|  | Education | .12 | .003 | - | - |
|  | COVID-19 index | -.07 | .081 | - | - |
|  | Affective well-being Baseline | .54 | .000 | - | - |
| **Cohort 2 [DV Cognitive well-being Follow-up]** | | | | | |
| 1 |  |  |  | .007 | .016 |
|  | Education | -.09 | .016 | - | - |
| 2 |  |  |  | .331 | <.001* |
|  | Education | -.05 | .099 | - | - |
|  | Affective well-being Baseline | .57 | .000 | - | - |

We ran a sensitivity analysis in a regression framework to test against alternative specifications with regard to affective and cognitive well-being. Firstly we added possible covariates to the regression, in a second step we added the baseline measure of well-being to the model. In both of the cohorts, the added value of baseline affective well-being was significant, above and beyond the covariates.

## Appendix A4. Description of adolescent groups of increased, decreased and similar affective well-being Cohort 1

|  | **Increased in affective well-being** | **Decreased in affective well-being** | **Similar affective well-being** |
| --- | --- | --- | --- |
|  | **Baseline**  **(*N=*207)** | **Baseline**  **(*N=*96)** | **Baseline**  **(*N=*159)** |
| **Demographics** | | | |
| Age | 16.54 (2.97) | 16.36 (3.32) | 15.87 (3.04) |
| Sex % girls | 78.7% | 76.0% | 65.4% |
| Education level | 1.0% primary school  14.5% low  22.7% medium  52.7% high | 4.2% primary school  12.5% low  21.9% medium  58.3% high | 2.5% primary school  11.3% low  22.6% medium  57.2% high |
| Cultural identity | 90.3% Dutch, 5.3% mixed, 1.0% other | 86.5% Dutch, 10.4% mixed, 0% other | 85.5% Dutch, 9.4% mixed, 1.9% other |
| **Mental health status** | | | |
| In treatment for psychological problems | 16.9%  (and 1.9% waitinglist) | 8.4%  (and 1.0% waitinglist) | 11.9%  (and 1.3% waitinglist) |
| Mode of contact since COVID-19 with psychologist/psychiatrist | 34.3% offline  62.9% online  2.9% none | 12.5% offline  87.5% online  0.0% none | 36.8% offline  57.9% online  5.3% none |
| Affective well-being | 4.23 (1.22) | 5.58 (1.15) | 5.39 (1.24) |
| Cognitive well-being | 5.77 (2.00) | 7.10 (2.04) | 7.25 (1.98) |
| Depressive symptoms | 6.56 (4.21) | 5.07 (3.93) | 4.33 (3.91) |
| Anxiety symptoms | 17.56 (4.40) | 15.87 (4.89) | 14.56 (4.29) |
| **Coping strategies** |  |  |  |
| Adaptive coping | 4.29 (1.39) | 3.97 (1.36) | 4.36 (1.29) |
| Maladaptive coping | 3.58 (1.69) | 3.16 (1.53) | 3.26 (1.46) |
| **Impact of COVID-19** | | | |
| Difficulties with cancelations | 2.58 (1.03) | 2.54 (0.99) | 2.35 (1.00) |
| Financial problems | 1.31 (0.63) | 1.16 (0.40) | 1.18 (0.54) |
| Optimism about the future | 2.09 (0.91) | 2.31 (0.92) | 2.32 (0.95) |
| Atmosphere at home | 4.92 (1.13) | 5.36 (1.17) | 5.55 (1.29) |
| COVID19 stringency index | 69.49 (3.45) | 70.69 (2.18) | 70.09 (2.94) |
| **User evaluation and engagement** |  |  |  |
| User evaluation of Grow It! app | 7.19 (1.43) | 6.88 (1.45) | 7.25 (1.47) |
| Challenges | 37.84% (29.57) | 42.62% (31.05) | 35.38% (29.71) |
| Compliance ESM | 23.47% (25.61) | 25.06% (25.06) | 20.27% (23.76) |

## Appendix A5. Description of adolescent groups of increased, decreased and similar cognitive well-being Cohort 1

|  | **Increased in cognitive well-being** | **Decreased in cognitive well-being** | **Similar cognitive well-being** |
| --- | --- | --- | --- |
|  | **Baseline**  **(*N=*244)** | **Baseline**  **(*N=*121*)*** | **Baseline**  **(*N=*97)** |
| **Demographics** | | | |
| Age | 16.46 (3.05) | 16.12 (3.11) | 15.99 (3.11) |
| Sex % girls | 73.8% | 77.7% | 68.0% |
| Education level | 0.9% primary school  12.9% low  26.2% medium  60.0% high | 5.3% primary school  20.4% low  23.9% medium  50.4% high | 2.2% primary school  8.7% low  19.6% medium  69.6% high |
| Cultural identity | 92.4% Dutch, 6.8% mixed, 0.8% other | 92.2% Dutch, 7.8% mixed, 0.0% other | 85.3% Dutch, 11.6% mixed, 3.2% other |
| **Mental health status** | | | |
| In treatment for psychological problems | 16.1%  (and 1.7% waitinglist) | 9.2%  (and 1.7% waitinglist) | 12.4%  (and 1.0% waitinglist) |
| Mode of contact since COVID-19 with psychologist/psychiatrist | 30.8% offline  66.7% online  2.7% none | 18.2% offline  81.8% online  0.0% none | 50.0% offline  41.7% online  8.3% none |
| Affective well-being | 4.58 (1.30) | 5.31 (1.28) | 5.24 (1.40) |
| Cognitive well-being | 5.78 (2.0) | 7.44 (1.94) | 7.40 (2.0) |
| Depressive symptoms | 6.08 (4.31) | 5.29 (3.95) | 4.24 (3.80) |
| Anxiety symptoms | 16.85 (4.52) | 15.55 (4.78) | 15.27 (4.61) |
| **Coping strategies** |  |  |  |
| Adaptive coping | 4.36 (1.30) | 3.89 (1.41) | 4.43 (1.35) |
| Maladaptive coping | 3.39 (1.60) | 3.34 (1.53) | 3.43 (1.63) |
| **Impact of COVID-19** | | | |
| Difficulties with cancelations | 2.54 (1.01) | 2.50 (1.07) | 2.38 (0.95) |
| Financial problems | 1.24 (0.55) | 1.22 (0.60) | 1.26 (0.55) |
| Optimism about the future | 2.02 (0.81) | 2.40 (0.99) | 2.47 (1.03) |
| Atmosphere at home | 5.08 (1.24) | 5.42 (1.09) | 5.38 (1.32) |
| COVID19 stringency index | 69.63 (3.35) | 70.47 (2.50) | 70.06 (2.95) |
| **User evaluation and engagement** |  |  |  |
| User evaluation of Grow It! app | 7.22 (1.46) | 7.05 (1.40) | 7.05 (1.50) |
| Challenges | 35.15% (28.58) | 39.40% (30.36) | 43.61% (32.36) |
| Compliance ESM | 21.42% (24.76) | 22.48% (23.43) | 26.46% (27.01) |

## Appendix A6. Description of adolescent groups of increased, decreased and similar affective well-being Cohort 2

|  | **Increased in affective well-being** | **Decreased in affective well-being** | **Similar affective well-being** |
| --- | --- | --- | --- |
|  | **Baseline**  **(*N=*307)** | **Baseline**  **(*N=*169*)*** | **Baseline**  **(*N=*257)** |
| **Demographics** | | | |
| Age | 18.42 (3.27) | 18.61 (3.52) | 18.45 (3.66) |
| Sex % girls | 85.5% | 77.2% | 79.0% |
| Education level | 1.0% primary school  15.0% low  31.7% medium  47.0% high  5.3% other | 2.4% primary school  13.2% low  30.5% medium  45.5% high  8.4% other | 0.0% primary school  16.7% low  31.1% medium  43.8% high  8.4% other |
| Cultural identity | 98.0% Dutch, 1.6% mixed, 0.3% other | 97.6% Dutch, 1.8% mixed, 0.6% other | 98.4% Dutch, 1.6% mixed, 0.0% other |
| **Mental health status** | | | |
| In treatment for psychological problems | 19.7%  (and 5.0% waitinglist) | 18.3%  (and 6.7% waitinglist) | 23.4%  (and 4.8% waitinglist) |
| Mode of contact since COVID-19 with psychologist/psychiatrist | 71.2% offline  25.4% online  3.4% none | 70.0% offline  30.0% online  0.0% none | 65.5% offline  32.8% online  1.7% none |
| Affective well-being | 3.67 (1.17) | 4.96 (1.30) | 4.53 (1.34) |
| Cognitive well-being | 4.97 (1.92) | 6.00 (2.17) | 5.86 (2.12) |
| Depressive symptoms | 7.86 (4.10) | 7.17 (4.40) | 6.92 (4.73) |
| Anxiety symptoms | 17.12 (4.11) | 18.91 (4.48) | 18.35 (4.71) |
| **Coping strategies** |  |  |  |
| Adaptive coping | 4.05 (1.27) | 4.21 (1.30) | 3.95 (1.28) |
| Maladaptive coping | 3.48 (1.48) | 3.67 (1.60) | 3.42 (1.56) |
| **Impact of COVID-19** | | | |
| Difficulties with cancelations | 2.67 (1.18) | 2.54 (1.20) | 2.46 (1.21) |
| Financial problems | 1.27 (0.65) | 1.20 (0.48) | 1.27 (0.59) |
| Optimism about the future | 1.75 (0.84) | 1.80 (0.91) | 1.84 (0.83) |
| Atmosphere at home | 4.86 (1.34) | 5.04 (1.41) | 5.03 (1.41) |
| COVID19 stringency index | 78.53 (2.67) | 78.35 (2.81) | 78.43 (2.31) |
| **User evaluation and engagement** |  |  |  |
| User evaluation of Grow It! app | 7.21 (1.27) | 7.03 (1.42) | 7.22 (1.32) |
| Challenges | 57.87% (32.49) | 57.34% (25.87) | 55.27% (30.86) |
| Compliance ESM | 38.68% (28.75) | 36.70% (25.87) | 36.35% (28.13) |

## Appendix A7. Description of adolescent groups of increased, decreased and similar affective well-being Cohort 2

|  | **Increased in affective well-being** | **Decreased in affective well-being** | **Similar affective well-being** |
| --- | --- | --- | --- |
|  | **Baseline**  **(*N=*331)** | **Baseline**  **(*N=*221*)*** | **Baseline**  **(*N=*181)** |
| **Demographics** | | | |
| Age | 18.34 (3.42) | 18.52 (3.54) | 18.71 (3.46) |
| Sex % girls | 83.5% | 77.3% | 82.1% |
| Education level | 1.2% primary school  17.0% low  28.7% medium  46.6% high  6.5% other | 1.4% primary school  12.4% low  27.2% medium  52.1% high  6.9% other | 0.0% primary school  15.3% low  40.7% medium  35.6% high  8.5% other |
| Cultural identity | 98.2% Dutch, 1.8% mixed, 0.0% other | 98.2% Dutch, 1.4% mixed, 0.5% other | 97.8% Dutch, 1.7% mixed, 0.6% other |
| **Mental health status** | | | |
| In treatment for psychological problems | 19.8%  (and 5.2% waitinglist) | 20.2%  (and 5.2% waitinglist) | 22.9%  (and 5.7% waitinglist) |
| Mode of contact since COVID-19 with psychologist/psychiatrist | 71.9% offline  28.1% online  0.0% none | 74.4% offline  23.3% online  2.3% none | 57.5% offline  37.5% online  5.0% none |
| Affective well-being | 4.02 (1.31) | 4.57 (1.40) | 5.69 (2.09) |
| Cognitive well-being | 4.82 (1.89) | 6.43 (2.04) | 5.69 (2.09) |
| Depressive symptoms | 7.57 (4.25) | 7.07 (4.50) | 7.39 (4.62) |
| Anxiety symptoms | 18.84 (4.33) | 18.79 (4.46) | 18.74 (4.57) |
| **Coping strategies** |  |  |  |
| Adaptive coping | 4.03 (1.22) | 4.17 (1.25) | 3.94 (1.41) |
| Maladaptive coping | 3.44 (1.55) | 3.51 (1.56) | 3.61 (1.49) |
| **Impact of COVID-19** | | | |
| Difficulties with cancelations | 78.63 (2.50) | 2.55 (1.18) | 2.32 (1.09) |
| Financial problems | 1.29 (0.64) | 1.19 (0.47) | 1.25 (0.64) |
| Optimism about the future |  |  |  |
| Atmosphere at home | 4.87 (1.38) | 5.08 (1.26) | 4.97 (1.12) |
| COVID19 stringency index | 78.63 (2.50) | 78.38 (2.63) | 78.21 (2.66) |
| **User evaluation and engagement** | 1.78 (0.82) | 1.80 (0.93) | 1.80 (0.82) |
| User evaluation of Grow It! app | 7.22 (1.35) | 7.01 (1.43) | 7.28 (1.12) |
| Challenges | 56.88% (31.94) | 55.01% (30.23) | 58.98% (30.64) |
| Compliance ESM | 38.26% (29.00) | 34.88% (26.97) | 38.94% 26.77) |
